# Machine Learning-Based predictive model for adolescent metabolic syndrome: Utilizing data from NHANES 2007–2016

**DOI:** 10.1038/s41598-025-88156-4

**Published:** 2025-01-25

**Authors:** Yu-zhen Zhang, Hai-ying Wu, Run-wei Ma, Bo Feng, Rui Yang, Xiao-gang Chen, Min-xiao Li, Li-ming Cheng

**Affiliations:** 1 Department of Anesthesiology and Surgical Intensive Care Unit, Kunming Children’s Hospital, Kunming, Yunnan China; 2 Department of Emergency, The First Affiliated Hospital of Kunming Medical University, Kunming, Yunnan China; 3 Department of Cardiac Surgery, Fuwai Yunnan Hospital, Chinese Academy of Medical Sciences/Affiliated Cardiovascular Hospital of Kunming Medical University, Kunming, Yunnan China; 4 Department of Clinical Laboratory, The Third Affiliated Hospital of Kunming Medical University, Kunming, Yunnan China

**Keywords:** Machine learning, Metabolic syndrome, Predictive model, Adolescents, NHANES, Health care, Diagnosis, Disease prevention, Public health, Medical research, Machine learning, Endocrinology, Endocrine system and metabolic diseases

## Abstract

Metabolic syndrome (Mets) in adolescents is a growing public health issue linked to obesity, hypertension, and insulin resistance, increasing risks of cardiovascular disease and mental health problems. Early detection and intervention are crucial but often hindered by complex diagnostic requirements. This study aims to develop a predictive model using NHANES data, excluding biochemical indicators, to provide a simple, cost-effective tool for large-scale, non-medical screening and early prevention of adolescent MetS. After excluding adolescents with missing diagnostic variables, the dataset included 2,459 adolescents via NHANES data from 2007–2016. We used LASSO regression and 20-fold cross-validation to screen for the variables with the greatest predictive value. The dataset was divided into training and validation sets in a 7:3 ratio, and SMOTE was used to expand the training set with a ratio of 1:1. Based on the training set, we built eight machine learning models and a multifactor logistic regression model, evaluating nine predictive models in total. After evaluating all models using the confusion matrix, calibration curves and decision curves, the LGB model had the best predictive performance, with an AUC of 0.969, a Youden index of 0.923, accuracy of 0.978, F1 score of 0.989, and Kappa value of 0.800. We further interpreted the LGB model using SHAP, the SHAP hive plot showed that the predictor variables were, in descending order of importance, BMI age sex-specific percentage, weight, upper arm circumference, thigh length, and race. Finally, we deployed it online for broader accessibility. The predictive models we developed and validated demonstrated high performance, making them suitable for large-scale, non-medical primary screening and early warning of adolescent Metabolic syndrome. The online deployment of the model allows for practical use in community and school settings, promoting early intervention and public health improvement.

## Introduction

Metabolic syndrome (MetS) is a comprehensive manifestation of a group of metabolic abnormalities characterized by insulin resistance, including abdominal obesity, hypertension, hyperglycemia, high serum triglycerides and low serum high-density-lipoprotein-cholesterol (HDL-C)^1^. In recent years, several epidemiological studies have shown that the prevalence of MetS in adolescents has increased significantly worldwide, reaching 3%−10% in developed regions^2,3^. This shows that the high prevalence of adolescent MetS has become an important public health problem that urgently needs to be solved.

Insulin resistance due to visceral adiposity is usually at the core of MetS. Visceral adipocytes release various inflammatory mediators and free fatty acids, such as tumor necrosis factor-alpha (TNF-α), interleukin-6 (IL-6), and adiponectin, which trigger a systemic inflammatory response and promote insulin resistance^4,5^. Moreover, high levels of free fatty acids (e.g. non-esterified fatty acids) entering the bloodstream cause mitochondrial dysfunction and oxidative stress, inhibiting insulin signaling^6^. In addition, oxidative stress and the action of inflammatory mediators can cause endothelial dysfunction and atherosclerosis, thereby increasing the risk of cardiovascular disease^7^.

MetS poses a serious threat to the physical and mental health of adolescents today. High blood pressure can lead to headaches, fatigue, and poor concentration, whereas obesity and high blood sugar significantly increase the risk of type 2 diabetes in adolescents^8,9^. Adolescents with MetS are also thought to be more likely to develop non-alcoholic fatty liver disease, which can lead to liver damage and dysfunction^10,11^. In addition, MetS has been associated with a range of mental health problems, and adolescents with this disease are more likely to experience psychological problems including depression and anxiety^12,13^. The impact of MetS on the health of adolescents in the future is even more profound. A history of metabolic syndrome in adolescence is a significant predictor of cardiovascular disease and type 2 diabetes in adulthood. This means that adolescents with MetS face greater risks of heart attack, stroke, and diabetes than adults do^14–16^. Additionally, studies have shown that MetS is associated with other chronic diseases in adulthood. For example, obesity and insulin resistance increase the risk of polycystic ovary syndrome and certain types of cancer, while the long-term systemic inflammation caused by MetS is also thought to underlie chronic diseases such as fatty liver and autoimmune disorders^17–20^.

If we can achieve early detection, early control and early treatment of metabolic syndrome in adolescents, we can significantly improve the current health level and quality of life of adolescent patients, and reduce their risk of more chronic diseases in the future.

Currently, several studies have developed predictive models for metabolic syndrome that include methods such as normograms and machine learning algorithms^21–23^. However, there are still several problems with these models. Some studies are based primarily on data from adults and lack consideration of the specific physiologic and metabolic characteristics of adolescents. Other studies include fewer variables or populations and have limited predictive power for metabolic syndrome. Existing diagnostic criteria often require blood tests and blood pressure measurements to obtain appropriate data. The basic equipment required for blood collection and testing, as well as blood pressure measurement, is usually only available in healthcare facilities, which is not conducive to large-scale rapid screening of populations in schools, neighborhoods, or even homes. The key point of MetS management is early detection and intervention, which may be delayed by having to visit a healthcare facility for screening. Therefore, we hope to find a simpler and easier way to obtain indicators, such as weight, length and other indicators, that can be objectively quantified, but can also be realized through simple operations, do not need special equipment, and are economical and convenient.

Therefore, we are committed to including a larger population of adolescent participants and more variable data from the National Health and Nutrition Examination Survey (NHANES). To develop a model that facilitates self-assessment of MetS risk in the youth population and their families and is deployed online as a web tool, after biochemical indicators are excluded to enable earlier prevention and intervention of metabolic syndrome in the youth population.

## Methods

### Data sources

**Fig. 1:**
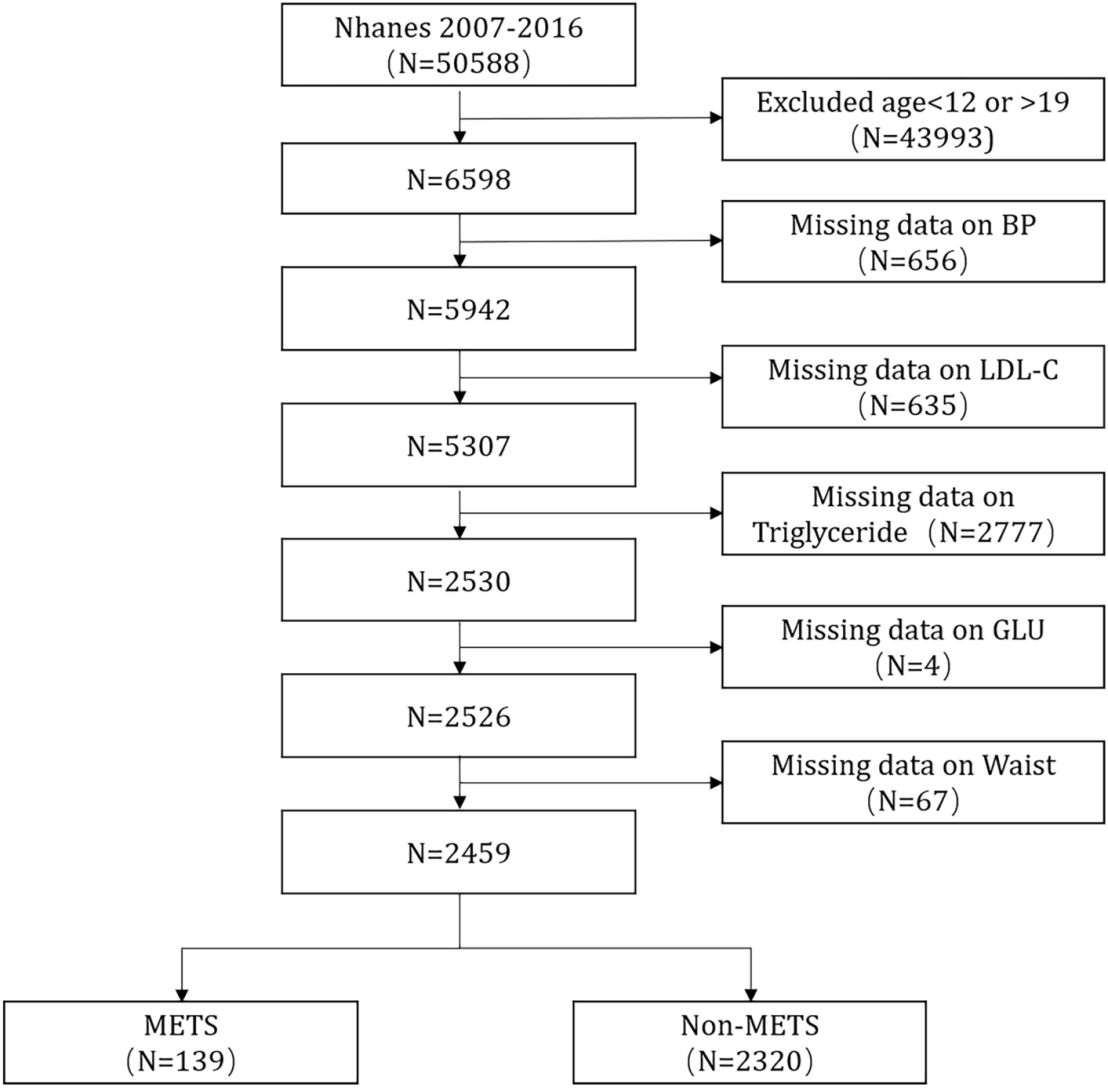
Flow chart for patient inclusion. Flow chart of the selection of participants from NHANES 2007–2016. BP, blood pressure; LDL-C, high density BP, blood pressure; LDL-C, high density lipoprotein cholesterol; GLU, fasting glucose.

The data for this study were derived from the NHANES, which is sponsored by the Centers for Disease Control and Prevention (CDC). The NHANES data are nationally representative through the design of a complex multistage sample. As shown in Fig. 1, we counted data from 5 cycles from 2007–2016, with a total of 50,588 participants. After excluding participants aged outside the range of 12 to 19 years and those whose waist circumference data, fasting glucose data, serum HDL data, triglyceride data, and blood pressure measurements were missing, 2459 eligible participants were included. Owing to missing NHANES data, we used the predictive mean matching (PMM) method for variables other than diagnostic variables to fill in the gaps based on data from other variables.

### Diagnostic

MetS in adolescents remains somewhat controversial, so we defined the diagnosis of MetS as meeting any three of the following five criteria, on the basis of the recommendations of the American Heart Association (AHA) and the National Heart, Lung, and Blood Institute (NHLBI)^24^, as well as the Comprehensive Guidelines for Cardiovascular Health and Risk Reduction in Young People^25^:

I. Waist circumference ≥ 102 cm in men and ≥ 88 cm in women; II. Serum triglycerides ≥ 130 mg/dl; III. HDL-C < 40 for men and < 50 for women; IV. Systolic blood pressure ≥ 130 mmHg or diastolic blood pressure ≥ 85 mmHg; V. Fasting blood glucose ≥ 5.6 mg/dl.

### Variable definition and variable selection

The variables we screened from the NHANES database consisted of four parts: I. Diagnostic variables of MetS. II. The participants’ demographic data included race, age, sex, the household poverty income ratio (PIR), family history of diabetes, physical activity, and sedentary time. III. Participant physical measurement data, including height, weight, body mass index (BMI), upper arm length, upper wall arm circumference, and thigh length. IV. Participant consumer behavior questionnaire data.

Physical activity was defined according to the NHANES questionnaire, and adolescents who performed less than 10 min of vigorous or moderate physical activity per week were defined as inactive. PIR was the ratio of household income to the poverty line, with ≤ 1 indicating that the participant's household was in poverty. The BMI sex-age-specific percentage (BMI_PERC) was defined according to the CDC2000 growth charts, which better reflects the physical development of adolescents.

### Baseline analysis

In the baseline analysis, considering the complex sampling design and varying sampling weights of NHANES, we applied appropriate NHANES weight settings to calculate the weighted means of continuous variables along with their corresponding standard errors, and determined the weighted percentage shares of categorical variables. Weighted t-tests and chi-square tests were then used to evaluate the differences in the distribution of continuous and categorical variables between the MetS group and the non-MetS group.

### Feature selection

We developed a Least Absolute Shrinkage and Selection Operator (LASSO) regression model to explore the relationships between all nonbiochemical indicator variables, excluding diagnostic variables, and the prevalence of MetS. LASSO regression controls the complexity of the model by introducing a regularization parameter, lambda. Through L1 regularization of the feature coefficients, LASSO regression can compress the coefficients of some features to zero, which enables feature selection. We subsequently optimized the LASSO model via 20-fold cross-validation and selected lambda.1se as the regularization parameter for the final model.

### Modeling

To build the MetS prediction model and test its predictive ability, we divided all the participant data into a training set and a validation set at a ratio of 7:3.

To solve the problem of category imbalance and improve model performance, we performed sample augmentation using the SMOTE algorithm on the training set data before model building, with an augmentation ratio of 1:1. After that, we used these variables as independent variables to build models based on the expanded training set data. Including Decision Tree, Random Forest (RF), Extreme Gradient Boosting (XGB), Gradient Boosting Machine (GBM), Light Gradient Boosting Machine (LGB), Support Vector Machine (SVM), Plain Bayes, and Multilayer Perceptron (MLP). In total, eight machine learning models and a multivariate logistic regression model were constructed.

### Model evaluation and interpretation

For the validation set, we constructed ROC curves and confusion matrices and evaluated the 9 models via the C-index, Youden's index, F1 scores, and Kappa scores to identify the model with the best discriminative power. We then plotted calibration curves and decision curves to further validate the predictive ability and application value of the models. The models were interpreted via Shapley Additive exPlanations (SHAP).

All analyses and modeling in this study were performed with the statistical software R 4.3.3 and RStudio. All tests were two sided and considered statistically significant with a p-value of < 0.05.

## Results

### Baseline analysis

Based on the diagnostic criteria, 139 out of 2459 adolescents from 5 NHANES-cycles were diagnosed with MetS, as detailed in Table 1. Compared with adolescents without metabolic syndrome, those with metabolic syndrome had a greater waist circumference (109.59 cm, P < 0.0001), upper arm length (38.15 cm, P < 0.0001), upper arm circumference (36.74 cm, P < 0.0001), age (16.03 years, P = 0.0103), percentage of family history of diabetes (23.78%, P = 0.0001), and BMI_PERC (0.97, P < 0.0001) values were significantly greater. In contrast, the PIR (1.98, P = 0.0046) was lower. There was also a significant difference in the racial distribution of patients in the two groups. In the MetS group, the proportion of Mexican Americans was significantly greater (28.24%), whereas the proportions of non-Hispanic blacks (9.92%), non-Hispanic whites (53.15%), and other ethnic groups (1.81%) were significantly lower.

**Table 1:** Basic characteristics of participants in the NHANES 2007–2016.

Variable		Total (n = 2459)			METS (n = 139)	Non-METS (n = 2320)	P- Value
Demographic data							
Age (RIDAGEYR)		15.46 ± 0.06			16.03 ± 0.23	15.43 ± 0.06	P = 0.0103
Gender (%) (RIAGENDR)							P = 0.2121
Male		52.16%			58.84%	51.79%	
Female		47.84%			41.16%	48.21%	
Race (%) (RIDRETH1)							P < 0.0001
Mexican American		13.86%			28.24%	13.08%	
Other Hispanic		6.95%			6.88%	6.96%	
Non-Hispanic White		57.37%			53.15%	57.60%	
Non-Hispanic Black		14.21%			9.92%	14.44%	
Other Race		7.60%			1.81%	7.92%	
PIR (INDFMPIR)		2.51 ± 0.07			1.98 ± 0.19	2.54 ± 0.07	P = 0.0046
Family history of diabetes (%) (DIQ170)							P = 0.0001
YES		9.44%			23.78%	8.66%	
NO		90.56%			76.22%	91.34%	
Biochemical index							
Triglyceride (LBXTR)		79.60 ± 1.37			159.4 ± 7.21	75.26 ± 1.32	P < 0.0001
HDL-C (LBDHDD)		52.56 ± 0.34			38.25 ± 0.83	53.34 ± 0.35	P < 0.0001
Fasting blood glucose (LBDGLUSI)		5.28 ± 0.02			5.61 ± 0.05	5.27 ± 0.02	P < 0.0001
Body measurement							
BMI_PERC		0.68 ± 0.01			0.97 ± 0.01	0.66 ± 0.01	P < 0.0001
High (BMXHT)		166.40 ± 0.27			168.60 ± 0.91	166.28 ± 0.28	P = 0.0134
Weight (BMXWT)		66.78 ± 0.54			97.46 ± 2.47	65.12 ± 0.50	P < 0.0001
Waist (BMXWAIST)		81.99 ± 0.42			109.59 ± 1.87	80.49 ± 0.41	P < 0.0001
Thigh length (BMXLEG)		40.00 ± 0.10			40.65 ± 0.32	39.96 ± 0.10	P = 0.0401
Upper arm length (BMXARML)		36.24 ± 0.08			38.15 ± 0.27	36.13 ± 0.08	P < 0.0001
Arm circumference (BMXARMC)		29.01 ± 0.15			36.74 ± 0.56	28.59 ± 0.14	P < 0.0001
Systolic blood pressure (BPXSY1)		109.47 ± 0.35			118.99 ± 1.25	108.95 ± 0.35	P < 0.0001
Diastolic blood pressure (BPXDI1)		59.45 ± 0.48			62.59 ± 1.17	59.28 ± 0.50	P = 0.0086
Sedentary time (PAD680)		461.77 ± 5.97			487.23 ± 17.59	460.38 ± 6.32	P = 0.1660
Activity							P = 0.3015
Active		78.91%			74.79%	79.13%	
Inactive		21.09%			25.21%	20.87%	
Consumer data							
Weekly fast food (DBQ900)		2.06 ± 0.08			2.16 ± 0.20	2.05 ± 0.09	P = 0.6058
Monthly ready-to-eat food (DBQ905)		1.72 ± 0.11			1.79 ± 0.55	1.72 ± 0.11	P = 0.9006
Monthly frozen food (DBQ910)		3.62 ± 0.22			2.98 ± 0.49	3.65 ± 0.23	P = 0.1964
Weekly meals not at home (DBQ895)		3.23 ± 0.13			3.00 ± 0.30	3.24 ± 0.13	P = 0.4237
Does the school provide breakfast (DBQ400)							P = 0.6904
YES		81.98%			83.64%	81.89%	
NO		18.02%			16.36%	18.11	
School breakfast prices (DBQ421)							P = 0.1374
Free		45.03%			57.66%	44.34%	
Reduced price		25.49%			21.33%	25.72%	
Full price		29.48%			21.01%	29.94%	
School breakfast several times a week (DBQ411)		1.20 ± 0.06			1.63 ± 0.18	1.18 ± 0.06	P = 0.0100
Does the school provide lunch (DBQ370)							P = 0.8092
YES		94.82%			94.24%	94.85%	
NO		5.18%			5.76%	5.15%	
School lunches several times a week (DBQ381)		3.18 ± 0.07			3.65 ± 0.18	3.15 ± 0.07	P = 0.0063
Summer program meal free/reduced price (DBQ424)							P = 0.5617
YES		5.61%			7.47%	5.51%	
NO		47.00%			42.72%	47.24%	
No summer program meal		47.39%			49.81%	47.25%	

Continuous variables are expressed as weighted mean ± SE, P-values were calculated using a weighted t-test. The P-values for categorical variables were calculated using weighted chi-square tests. In parentheses for each variable name is the corresponding NHANES code. Abbreviations: NHANES (National Health and Nutrition Examination Survey), PIR (Poverty Ratio of Household Income), BMI (Body Mass Index), BMI_PERC (BMI Age-Sex-Specific Percentage), HDL-C (High Density Lipoprotein Cholesterol)

### Predictor screening and modeling

**Fig. 2:**
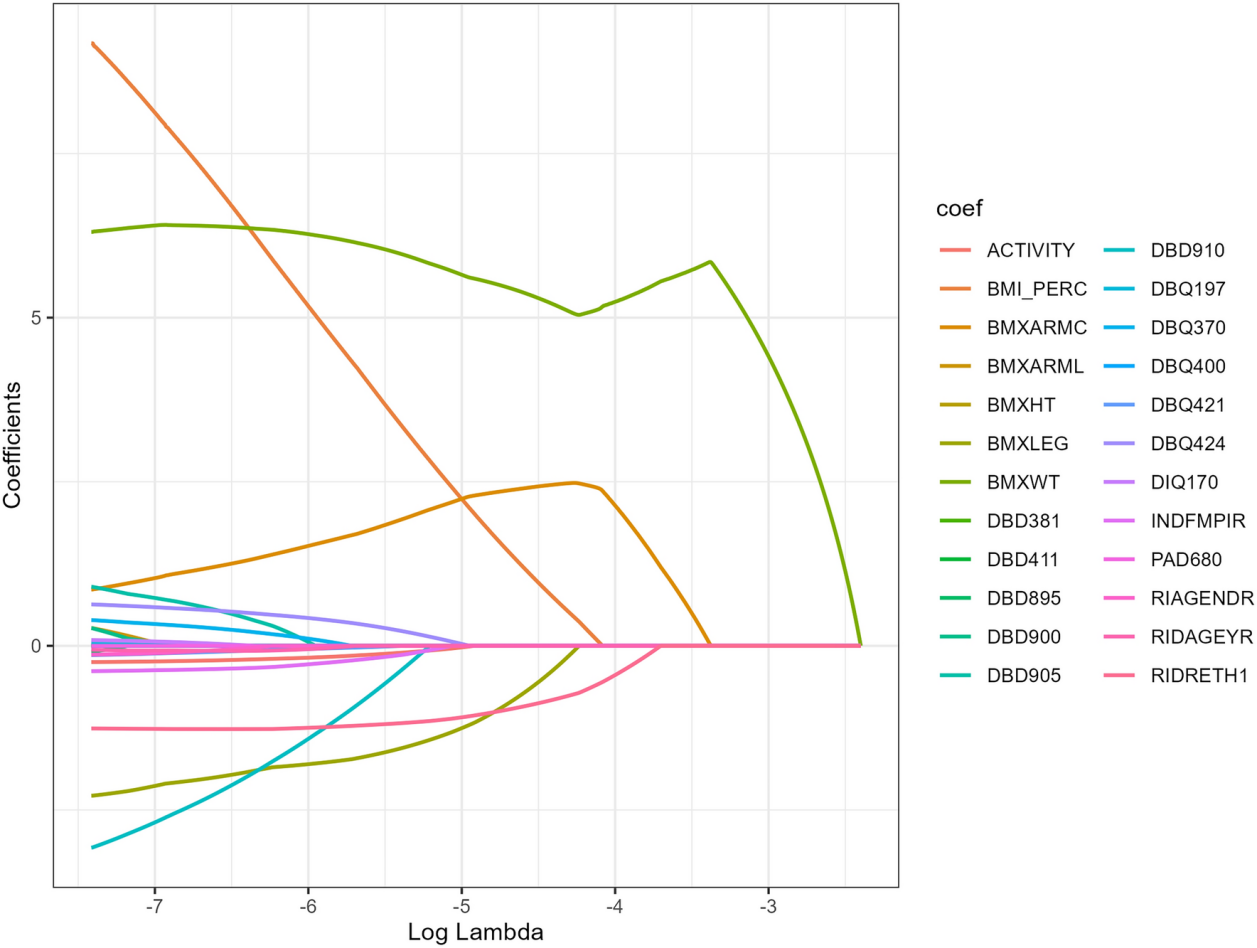
Path diagram for selecting variables for the LASSO regression model. The horizontal axis is the logarithmized penalty parameter log(λ), and the vertical axis is the corresponding regression coefficient value. The horizontal axis is the logarithmized penalty parameter log(λ), and the vertical axis is the corresponding regression coefficient value. As the penalty parameter increases, the regression coefficients of each variable will eventually approach zero, and thus, as the penalty parameter increases, the regression coefficients of each variable will eventually approach zero; LASSO regression can filter out the variables with the greatest predictive value. Variable coding is from the NHANES.

**Fig. 3:**
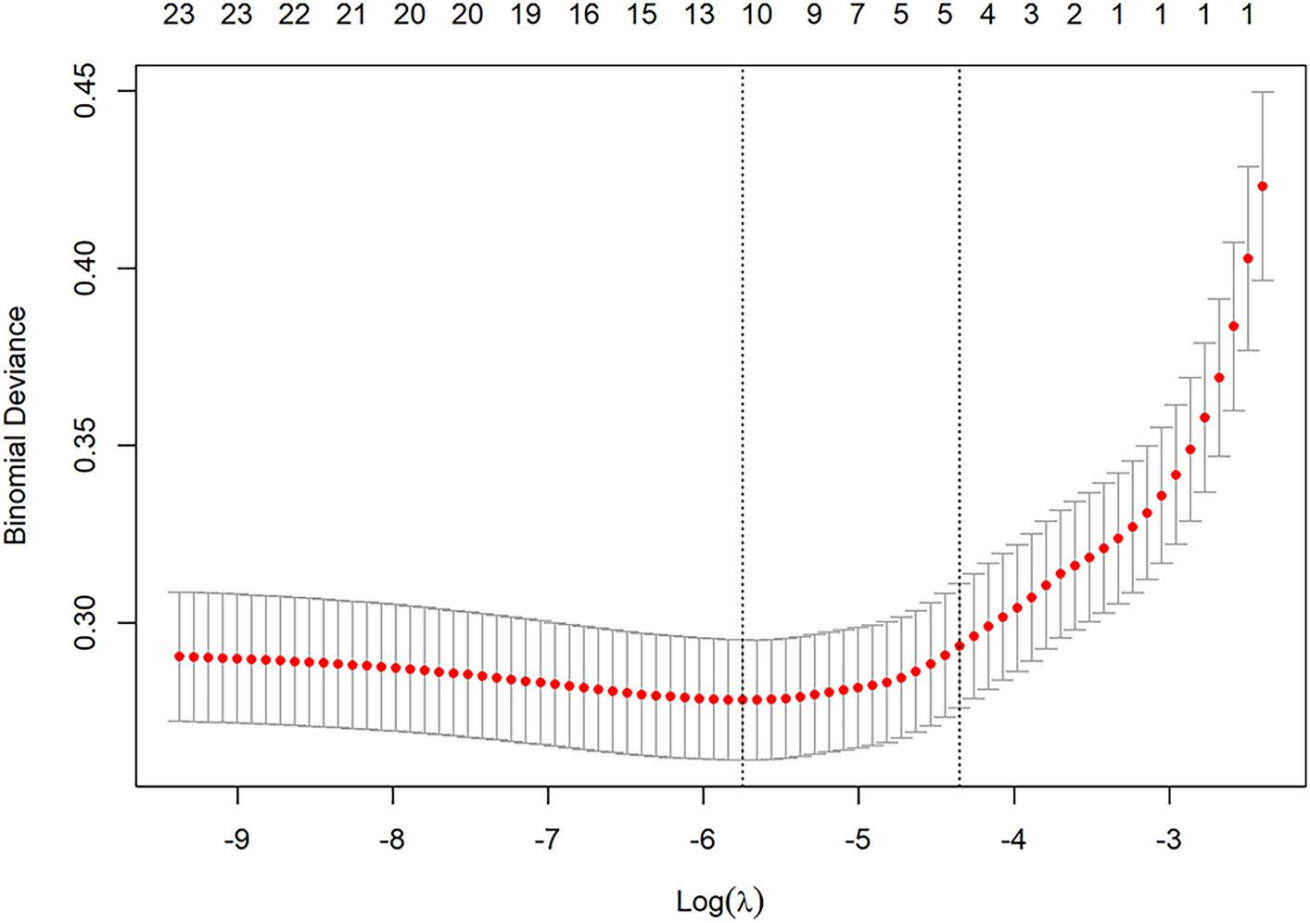
Results of the 20-fold cross-validation of the LASSO regression model. The horizontal axis is the penalty parameter log (λ), and the vertical axis is the binomial deviation. The red solid dots represent each λ value corresponding to the mean deviation, and the vertical gray error bars indicate the standard error of that deviation. The red solid dots represent each λ value corresponding to the mean deviation, and the vertical gray error bars indicate the standard error of that deviation. The red solid dots represent each λ value corresponding to the mean deviation, and the vertical gray error bars indicate the standard error of that deviation. The upper numbers indicate the number of nonzero coefficients under each value. This figure is used to evaluate the performance of the model at different values of λ and to select the model for each value. This figure is used to evaluate the performance of the model at different values of λ and to select the corresponding variables for model construction.

After excluding laboratory biochemical indicators and diagnostic criteria, we built a LASSO regression model and performed 20-fold cross-validation, and the results are shown in Figs. 2 and 3. We selected lambda.1 se and screened a total of five highly effective predictors of whether MetS was prevalent, including BMI_PERC, weight, upper arm circumference, thigh length and race.

The overall data were divided into a training set and a testing set at a ratio of 7:3. The difference in the data distributions between the two datasets is demonstrated in Table 2. Because the prevalence of MetS was not high in the original dataset, differences in the distribution of race variables became evident after the division. The remaining variables did not significantly differ in distribution between groups.

**Table 2:** Comparison of the data distributions between the training and test sets.

Variable	Train	Test	P-value
Weight	66.63 ± 0.48	67.04 ± 0.71	P = 0.2531
Percentage of BMI	0.68 ± 0.01	0.7 ± 0.01	P = 0.2672
Thigh length	39.97 ± 0.08	39.89 ± 0.13	P = 0.4106
Arm circumference	29.01 ± 0.13	29.13 ± 0.19	P = 0.2731
Race (%)			P = 0.0055
Mexican American	21.84%	27.14%	
Other Hispanic	11.27%	11.94%	
Non-Hispanic White	27.99%	29.04%	
Non-Hispanic Black	26.48%	22.93%	
Other Race	12.43%	22.93%	
MetS			P = 0.5883
Yes	5.46%	6.11%	
No	94.54%	93.89%	

Mean ± standard error for continuous variables: p-values were calculated using the t-test. Percentage of each category for categorical variables: p-value calculated by chi-square test

### Model evaluation

**Fig. 4:**
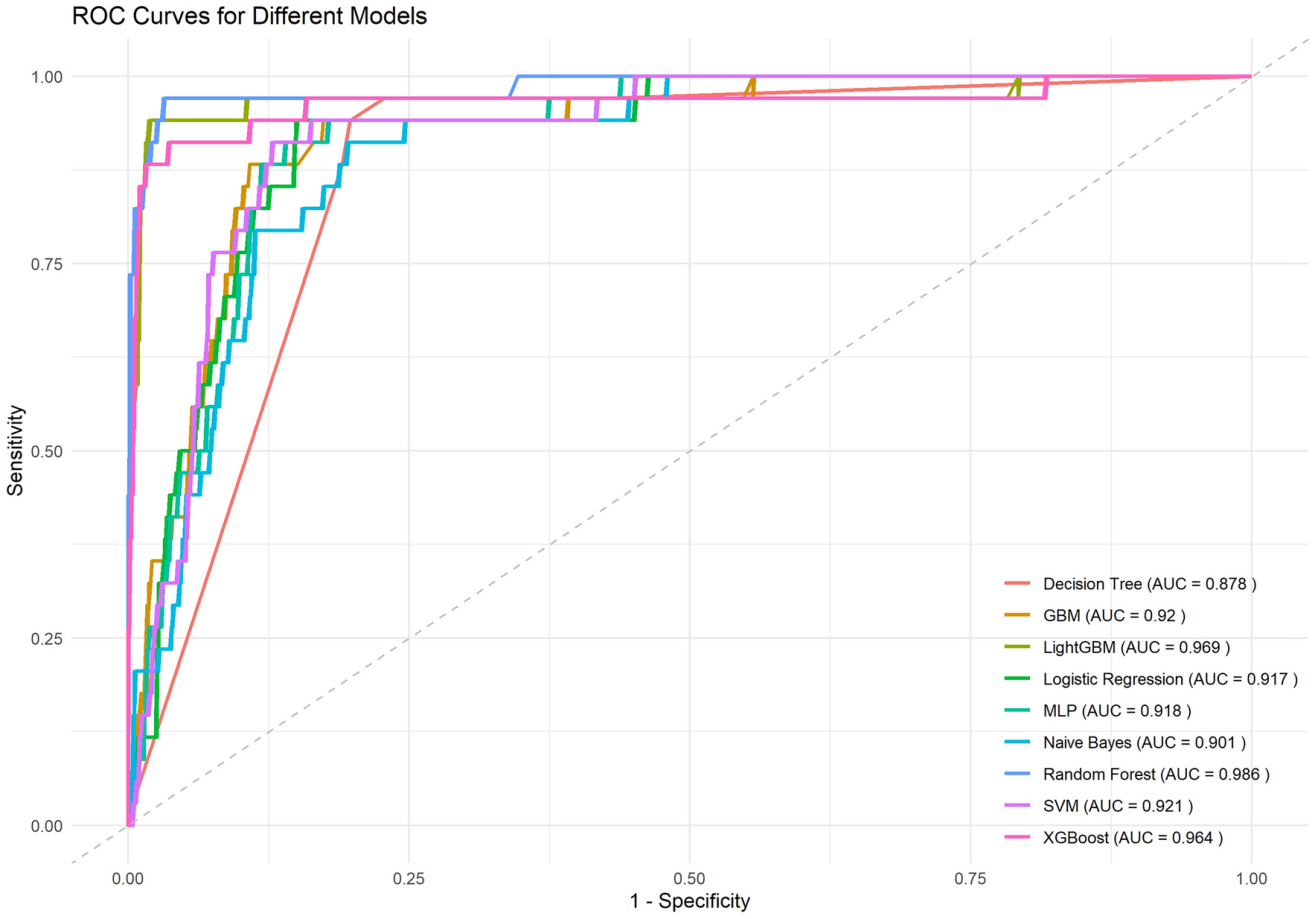
Receiver operating characteristic curves for 9 models. The values of the receiver operating characteristic curve (ROC) and its corresponding area under the curve (AUC) for nine different machine learning models. The horizontal axis is 1-Specificity, and the vertical axis is Sensitivity. The closer the curve is to the upper left corner, the better the model's classification performance.

We plotted the ROC curve (Fig. 4) and built a confusion matrix based on the validation set data, and the results are shown in Table 3. After combining the Youden index, AUC, accuracy, F1 score, and Kappa score, we comprehensively evaluated the model performance and concluded that the LGB model had the best model performance.

**Table 3:** Model performance table for 9 models.

Model	AUC	Youden's	Cutoff	Accuracy	Sensitivity	Specificity	F1	Kappa	NPV	PPV
Logistic	0.917	0.792	0.531	0.855	0.851	0.941	0.918	0.324	0.236	0.997
RF	0.986	0.940	0.368	0.969	0.969	0.971	0.983	0.726	0.600	0.999
SVM	0.921	−0.784	0.377	0.126	0.128	0.088	0.218	−0.086	0.005	0.744
XGB	0.964	0.876	0.246	0.962	0.964	0.912	0.980	0.670	0.554	0.996
GBM	0.920	0.774	0.621	0.891	0.892	0.882	0.940	0.386	0.283	0.994
LGB	0.969	0.923	0.528	0.978	0.982	0.941	0.989	0.800	0.711	0.997
NB	0.901	0.717	0.579	0.810	0.805	0.912	0.890	0.249	0.185	0.995
Tree	0.878	0.743	0.521	0.809	0.802	0.941	0.889	0.255	0.187	0.996
MLP	0.918	0.773	0.646	0.863	0.861	0.912	0.923	0.332	0.240	0.995

AUC, area under the curve; Youden's index, denotes the combined performance of the model, calculated as Sensitivity + Specificity – 1; Cutoff, value is the threshold for the model to classify the positive and negative classes; F1, harmonized average of precision and recall rates; Kappa, the agreement between the predictions of the classification model and the randomized prediction results; NPV, the negative predictive value, which indicates the proportion of predicted negative samples that are truly negative; PPV , the Positive Predictive Value, which indicates the proportion of samples predicted to be positive that are truly positive

**Fig. 5:**
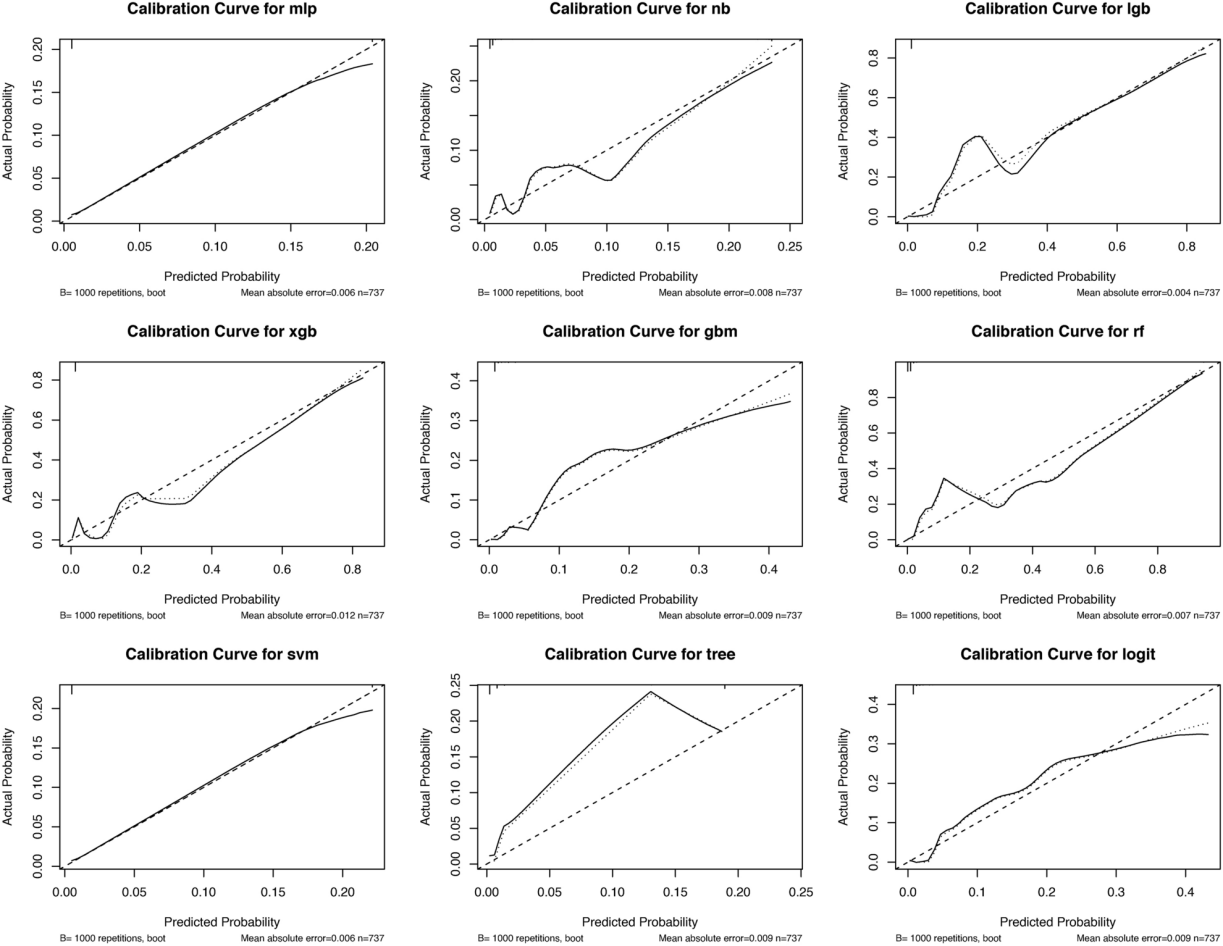
Calibration curves for 9 predictive models. The horizontal axis (X-axis) represents the probability of illness predicted by the model, and the vertical axis (Y-axis) represents the actual observed proportion of illness.

After that, we plotted the calibration curves of each model, and the results are shown in Fig. 5. The results show that the calibration curves of the models are incomplete except for three models, XGB, LGB and Random Forest. Among them, the LGB model mean absolute error (MAE) is the lowest (0.004), the Random Forest model is second (0.007), and the XGB model is the highest (0.012). However, the MAEs of all three models are lower than 0.05, which proves that the models have relatively good predictive ability and less deviation from the actual probability.

**Fig. 6:**
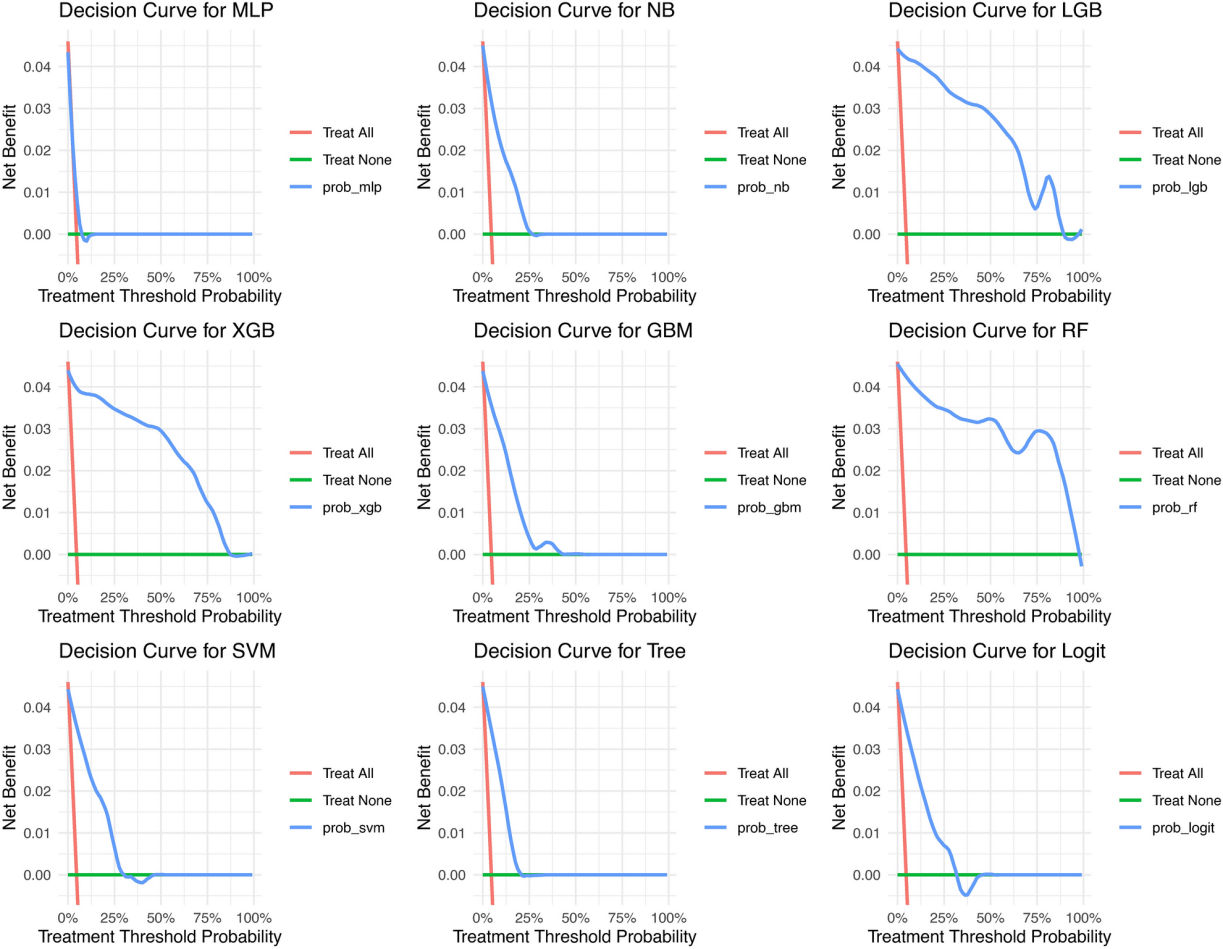
Decision curves for nine forecasting models. The horizontal axis (X-axis) represents the threshold probability of the model prediction, i.e., individuals with a predicted probability greater than this threshold are categorized as positive. The vertical axis (Y-axis) represents Net Benefit, whose value combines true positives and false positives predicted by the model. The “All” line (All): scenario in which all patients are assumed to receive treatment. The “No Treatment” line (None): a scenario in which all patients are assumed to receive no treatment.

In addition, we plotted the decision curve. The results are shown in Fig. 6, where the LGB model, the XGB model, and the Random Forest model all yield high net benefits under a wider range of thresholds, with significant clinical decision benefits. In summary, our evaluation results demonstrate that our machine learning model can identify adolescent MetS patients well and benefit them.

**Fig. 7:**
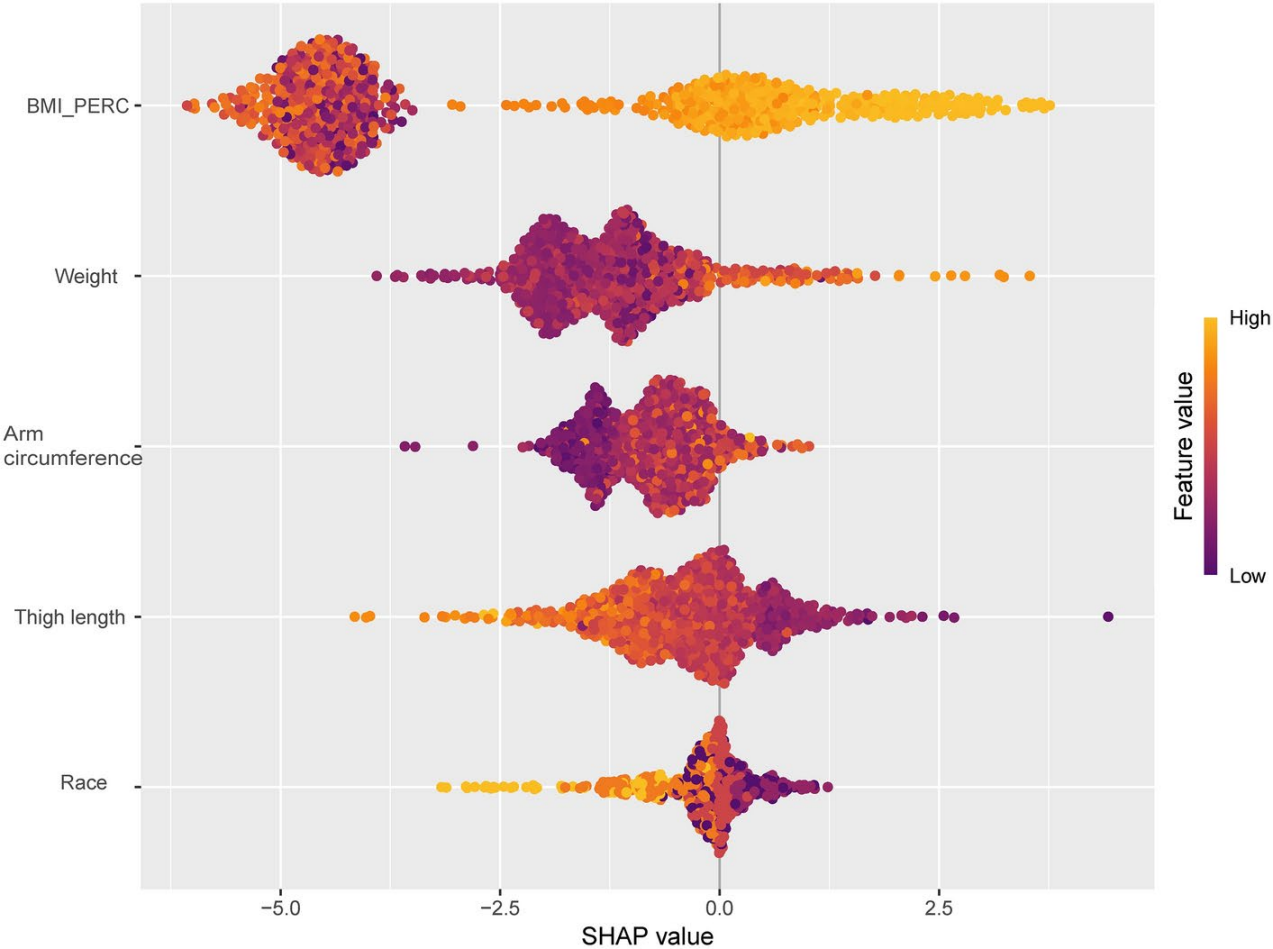
SHAP Beehive Diagrams for Random Forest Models. The horizontal axis (X-axis) represents the SHAP value (how much the feature affects the prediction) for each sample. The vertical axis (Y-axis) lists the important features in the model, which are sorted from top to bottom in order of importance. Each point represents the SHAP value for one sample, where yellow dots indicate high individual values and blue dots indicate low individual values.

Finally, we plotted SHAP hive plots (Fig. 7) showing the effects of different features on the model output. BMI_PERC had the greatest impact on model output, with a high percentage of individuals contributing positively towards the predictions; the higher the percentage was, the greater the contribution. Weight (BMXWT) and arm circumference (BMXARMC) subsequently contributed negatively to the prediction, mainly in individuals with low values. Thigh length (BMXLEG) had the fourth highest effect on model output, with high value individuals providing primarily negative contributions, whereas low value individuals provided positive contributions. Race contributed the least to model predictions, with non-Hispanic blacks and other races providing predominantly negative contributions and Mexican Americans and other Hispanics providing predominantly positive contributions.

### Online deployment of the model

Finally, to utilize our predictive model in real-world scenarios, we performed an online deployment of the LGB model^26^.

## Discussion

Our study analyzed five cycles of NHANES data, covering all adolescent participants during the period, with the aim of building reliable and robust predictive models of adolescent MetS. We built LASSO regression models using a total of 27 variables across three dimensions: demographic data, physical measurements, and consumer behavior questionnaire data. A machine learning model was also built using significant variables screened by 20-fold cross-validation, allowing the model to capture complex interactions between variables at multiple levels.

The effective identification of predictors is key to establishing an early prediction mechanism for MetS in high-risk adolescents. After excluding diagnostic criteria and biochemical indicators, by building a LASSO regression model and cross-validation with lambda.1 se, we screened five body measures with significant predictive value for MetS, including BMI_PERC, body weight, upper arm circumference, thigh length, and ethnicity.

Our study revealed that the racial distribution of MetS patients is dramatically different from that of normal adolescents and has a significant role in the prediction of MetS. Racial differences are reflected in genetics, culture, dietary habits and lifestyles, which affect metabolic health. Some studies have shown that certain races, such as Mexican Americans and African Americans, are more likely to develop MetS^27^. This may be related to genetic susceptibility, dietary habits, the living environment, and culture. For example, Mexican–American and African-American children have significantly greater intakes of high-sugar beverages and fast food than other races, and these dietary habits are associated with a greater risk of obesity and MetS^28^.

In addition, body weight, BMI_PERC and upper arm circumference serve as indicators of the degree of abdominal obesity and visceral fat accumulation in participants, and the core of metabolic syndrome is usually insulin resistance caused by visceral fat. They have been well documented in previous studies as key predictors of MetS^27,28^. However, thigh length is generally not considered a traditional indicator of metabolic health. It has been suggested that longer thigh length may be associated with greater muscle mass and lower levels of visceral fat, and that these factors all contribute to a lower risk of MetS^29,30^. In our study, thigh length, as a new indicator, played an important role in the prediction of MetS.

After a side-by-side comparison, we found that the LGB model (AUC: 0.969, Youden's: 0.923, Accuracy: 0.978, F1: 0.989, Kappa: 0.800) had the best predictive performance. Subsequent evaluation of the plotted calibration curves and decision curves indicated that the LGB model, as a tool with strong identification ability, can effectively identify the group of adolescents at high risk of MetS and help for further early intervention. We also plotted SHAP hive plots to interpret the random forest model, and the five variables were ranked in descending order of importance: BMI_PERC, weight, upper arm circumference, thigh length, and race. The first three of these predictors all showed positive correlations with the probability of developing the disease in the model, whereas thigh length, in contrast, showed a predominantly negative correlation, i.e., the longer the thigh was, the lower the likelihood of developing MetS. Finally, we deployed the model online for social use.

In addition, the model performance of the support vector machine model was poor. This may be due to the lower probability of occurrence of MetS in adolescents, leading to a category imbalance in the validation set. This bias ultimately led to poor results for the support vector machine model on performance data other than the AUC.

Through in-depth analysis of NHANES data from 2007–2016, this study successfully developed a predictive model for MetS in adolescents, based on nonbiochemical indicators, and demonstrated its robust predictive performance. The key point of the study is that we excluded all the indicators that require instruments for measurement, such as biochemical indicators and blood pressure, which results in the process of obtaining indicators without expensive laboratory equipment, reducing the cost of testing, and making it overall simpler and more efficient. However, it is important to note that the model is primarily applicable to populations resembling those in the NHANES dataset and may require further validation in other demographic or epidemiologic contexts. While this method is particularly suitable for large-scale screening and surveillance in public health systems and in settings such as neighborhoods, schools, and homes, future studies are needed to evaluate its accuracy and generalizability across diverse populations and settings.

Based on the findings of this study, policy recommendations can be made to public health and education authorities, such as the introduction of regular health screening in schools, the inclusion of simple physical measures, and the early identification and warning of adolescents at high risk for MetS, to provide a scientific basis for more effective public health policies. In addition, the results of this study can be utilized to carry out adolescent health education and publicity activities to increase public awareness of and attention to MetS. The health awareness and self-management ability of adolescents and parents can be enhanced through a variety of methods, such as scientific lectures, health promotion and interactive experiences.

Nevertheless, our study has several limitations. First, our data were derived from the NHANES, in which the questionnaire data relied on participants' self-reports, which may affect the accuracy of the data. Second, the NHANES data are cross-sectional and do not provide evidence of causality, and the lack of longitudinal data limits an in-depth understanding of the developmental process of MetS. Third, although we included a wide range of variables, we may still have missed some important underlying factors because MetS is influenced by numerous factors, such as psychological stress and genetic factors. Therefore, further localized data are needed to validate the model and consider more predictor variables. Fourth, although machine learning models such as LGB excel in predictive performance, their complexity may lead to a reduction in the model's explanatory power.

To address the limitations of the current study, future research should focus on the following areas. First, the inherent limitations of cross-sectional designs restrict causal inference and hinder the model's ability to predict the dynamic progression of diseases. Although we integrated multiple cycles of NHANES data and applied sample weighting adjustments to ensure the representativeness of the data, these methods cannot fully overcome the inherent shortcomings of cross-sectional data. Future research could adopt Inverse Probability Weighting (IPW) to reduce the influence of confounding factors by adjusting sample weights, thereby improving the model's causal inference ability. Additionally, studies have shown that combining bootstrap simulation and SHAP techniques can enhance model interpretability and help explore causal relationships between variables, which may support overcoming the limitations of cross-sectional data^31,32^. Moreover, combining longitudinal data with time-series models can provide deeper insights into the dynamic development and potential causal relationships of MetS.

To further validate the model's broad applicability, external validation studies using multi-center datasets (e.g., the UK Biobank and the China Health and Nutrition Survey (CHNS)) are necessary. Exploring adjustments to prediction thresholds for different epidemiological contexts is also crucial. Additionally, adopting feature analysis methods could help identify key predictors that perform consistently across different populations, thereby improving the model’s generalizability^33^.

Another important area for improvement is the specificity of predictive factors. While this study intentionally excluded biochemical indicators to improve practicality, future research could integrate simplified biochemical markers, such as the triglyceride-glucose index (TyG), which could provide a more comprehensive characterization of MetS's metabolic features^34^. Therefore, future studies should consider incorporating simplified biochemical markers to provide a layered model that balances practicality and accuracy, meeting the needs of diverse resource settings while ensuring precise identification and early intervention of high-risk individuals.

Finally, as demonstrated in biological annotation research, iterative optimization strategies help balance model performance and interpretability and support application in low-resource settings, such as through offline versions or mobile applications^35^. These efforts will lay a solid foundation for future research, enabling us to further optimize model performance, improve its applicability, and promote its widespread use in public health and clinical screening.

## Conclusions

We analyzed data from the 2007–2016 NHANES and used LASSO regression and 20-fold cross-validation to filter out demographic and body measurements with significant predictive power, and ultimately developed a machine-learning model of LGB based on non-biochemical indicators. The results of the model evaluation indicate that the model has relatively excellent predictive performance and has the potential for a wide range of applications. In addition, we interpreted the model using SHAP to increase the readability of the model and make it more convincing. Finally, the model was deployed as a web tool to enable large-scale non-medical primary screening and early warning of METS in adolescents. Future studies will use localized data to further optimize the model and improve its applicability in different settings, providing important support for early identification and intervention of METS.

## Data Availability

The data used in this study were obtained from NHANES, a national cross-sectional survey administered by the CDC to assess the health and nutritional status of the U.S. The NHANES ensures that the data collected are nationally representative by means of a complex multistage sampling design. Detailed statistics can be found at https://www.cdc.gov/nchs/nhanes/.

## Abbreviations

MetS: Metabolic Syndrome
NHANES: National Health and Nutrition Examination Survey
HDL-C: High-Density-Lipoprotein-Cholesterol
TNF-α: Tumor necrosis factor-alpha
IL-6: Interleukin-6
CDC: Centers for Disease Control and Prevention
AHA: American Heart Association
NHLBI: National Heart, Lung, and Blood Institute
PIR: Poverty Income Ratio
BMI: Body Mass Index
BMI_PERC: BMI sex-age-specific percentage
PMM: Predictive mean interpolation
LASSO: Least Absolute Shrinkage and Selection Operator
SHAP: SHapley Additive exPlanations
ROC: Receiver Operating Characteristic Curve
AUC: Area Under the Curve
MAE: Mean Absolute Error
XGB: EXtreme Gradient Boosting
GBM: Gradient Boosting Machine
LBG: Light Gradient Boosting Machine

## Declarations

### Duplicate publication

This manuscript has not been submitted or published elsewhere in part or in full.
